# Development of the pharmacogenomics and genomics literacy framework for pharmacists

**DOI:** 10.1186/s40246-021-00361-0

**Published:** 2021-10-16

**Authors:** Azhar T. Rahma, Iffat Elbarazi, Bassam R. Ali, George P. Patrinos, Luai A. Ahmed, Mahanna Elsheik, Fatma Al-Maskari

**Affiliations:** 1 Institute of Public Health, College of Medicine and Health Sciences, United Arab Emirates University, P.O. Box 17666, Al Ain, Abu Dhabi UAE; 2 Department of Genetics and Genomics, College of Medicine and Health Science, United Arab Emirates University, P.O. Box 17666, Al Ain, Abu Dhabi UAE; 3 Zayed Center for Health Sciences, United Arab Emirates University, P.O. Box 17666, Al Ain, Abu Dhabi UAE; 4 Department of Pharmacy, School of Health Sciences, University of Patras, 26504 Patras, Greece

**Keywords:** Framework, Genomics, Pharmacogenomics, Pharmacists, United Arab Emirates, Knowledge, Attitude, Skills, Literacy

## Abstract

**Background:**

Pharmacists play a unique role in integrating genomic medicine and pharmacogenomics into the clinical practice and to translate pharmacogenomics from bench to bedside. However, the literature suggests that the knowledge gap in pharmacogenomics is a major challenge; therefore, developing pharmacists’ skills and literacy to achieve this anticipated role is highly important. We aim to conceptualize a personalized literacy framework for the adoption of genomic medicine and pharmacogenomics by pharmacists in the United Arab Emirates with possible regional and global relevance.

**Results:**

A qualitative approach using focus groups was used to design and to guide the development of a pharmacogenomics literacy framework. The Health Literacy Skills framework was used as a guide to conceptualize the pharmacogenomics literacy for pharmacists. The framework included six major components with specific suggested factors to improve pharmacists’ pharmacogenomics literacy. Major components include individual inputs, demand, skills, knowledge, attitude and sociocultural factors.

**Conclusion:**

This framework confirms a holistic bottom-up approach toward the implementation of pharmacogenomics. Personalized medicine entails personalized efforts and frameworks. Similar framework can be created for other healthcare providers, patients and stakeholders.

## Introduction

Pharmacogenomics (PGx) is a rapidly growing field of personalized medicine focusing on the effect of genetic variation in drug response [1, 2]. It aims to improve drug efficacy and minimize adverse drug reactions (ADRs) [3–5]. ADR is defined as “any response to a drug which is noxious and unintended, and which occurs at doses normally used in man for prophylaxis, diagnosis, or therapy of disease, or for the modification of physiological function. [6]” For example, several retrospective and prospective studies show a strong association between the presence of HLA-B*57:01 allele and abacavir-induced hypersensitivity reaction [7–9]. Therefore, screening for the allele is highly recommended according to the FDA-approved drug label before administering the medication [10].

Prescribing the right drug with the right dose to the patient from the first time is the anchor of PGx and personalized medicine [11]. The American Society of Health-System Pharmacists (ASHP) highlighted, emphasized and detailed the responsibilities, roles and functions of the pharmacists in the PGx era [12]. Pharmacists lead a unique role in integrating PGx into the clinical practice and translate PGx from bench to bedside. This role has been extensively investigated in research and assessed in many models in the pragmatic field of oncology, cardiology, psychiatry, geriatric and pediatric medicine [13–21]. Nevertheless, pharmacists are lagging in fostering and implementing their anticipated role.

Studies show that pharmacists are faced with a plethora of needs, challenges and barriers that obstruct them from rising up to expectations. The literature emphasizes pharmacogenomic knowledge gap as a major challenge. In a study that interviewed pharmacists by Berenbrok et al. [22], participants stressed the importance of training, solid clinical resources and gaining access to PGx specialists to bridge the gap in their current knowledge of PGx.

Numerous studies have concluded that PGx literacy is the most cited barrier by pharmacists for the full implementation of PGx in the health setting; most of the pharmacists did not thoroughly study PGx during their coursework [23–25]. A study by Romagnoli et al. [26] stated that pharmacists demanded designing PGx tools as a vital step to facilitate the implementation of PGx in their workplace. Furthermore, in our assessment of the PGx knowledge of healthcare providers in the United Arab Emirates (UAE), respondents in our cohort echoed the same call [27].

Pharmacists strive for the synthesis of a framework to tackle their PGx literacy to facilitate the full implementation of PGx. The literature supports this—an article by Wurcel et al. [28] called for a model and framework to bridge the gap and smooth the implementation of diagnostic information in the era of personalized medicine.

There is conflict among researchers about the definition of health literacy. For the PGx literacy, we define it based on Baker [29] as “the dynamic skills needed to work in the health care setting.” Baker (2006) states that health literacy is context specific and fluctuates depending on the type of health problem, provider, and setting. Also, genetic literacy has been defined as “adequate understanding and awareness of a genomics foundation to permit knowledgeable outcomes on genetic issues” [30].

The Health Literacy Skills framework (HLS) of Squiers et al. [31] is a comprehensive framework that appreciates the dynamic nature of knowledge and skills. The HLS has been conceptualized based on existing theoretical concepts and evolved by addressing the limitation of these concepts. The HLS framework is targeted toward patients and individuals, and it ties health literacy with health outcomes. Moreover, it factors the influence of society, culture, family, media and infrastructure on health literacy. The HLS framework can steer interventions endeavoring health literacy. It is structured upon four main pillars: individual factors that impact literacy skills, stimuli for literacy, skills to understand and act upon stimuli and lastly mediators, facilitators and influences.

However, limited studies propose a personalized framework for pharmacists and other healthcare providers addressing their PGx literacy. In this article, the pharmacogenomics/genomics literacy framework for pharmacists (PGLP) is introduced—a personalized literacy framework for the adoption of PGx by healthcare providers concerned with genomics in UAE with possible regional and global relevance. This framework will guide stakeholders in their mission of equipping pharmacists, genetic counselors, doctors and nurses with skills required for the adoption and implementation of PGx.

## Materials and methods

The HLS framework captures a holistic approach toward literacy and takes into account individual and sociocultural influences; therefore, it was utilized to conceptualize the PGLP framework [31]. Moreover, it was used as a guide to develop a personalized framework to be used among pharmacists. A qualitative method was used to design and guide the formation of the PGLP literacy framework. Four focus group discussions were organized with inpatient, outpatient, clinical and resident pharmacists working in the UAE. The interview guide was structured based on the HLS framework. The guide explored PGx knowledge, attitude and practice of pharmacists and the future of genomics and PGx fields. All discussions were coded and intercoder reliability was ensured. NVivo 12 software was used to extract themes and visualize the findings. Detailed methodology about recruitment and logistics is available in our previous research [27]. This study was approved by Social Science Research Ethics Committee of United Arab Emirates University ERS_2017_5671.

## Findings

The pharmacogenomics/genomics literacy framework for pharmacists (PGLP) were conceptualized based on the themes, codes and associations extracted and analyzed from the focus group discussions [27].

**Fig. 1:**
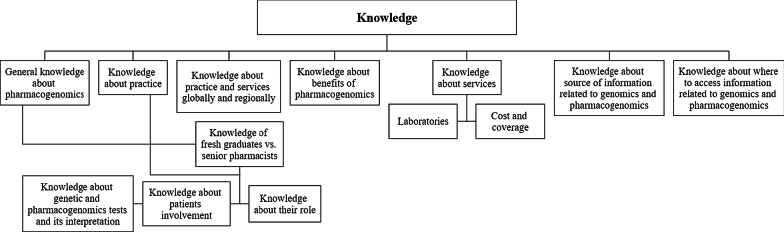
Main themes of PGx knowledge of pharmacists in the FGD

**Fig. 2:**
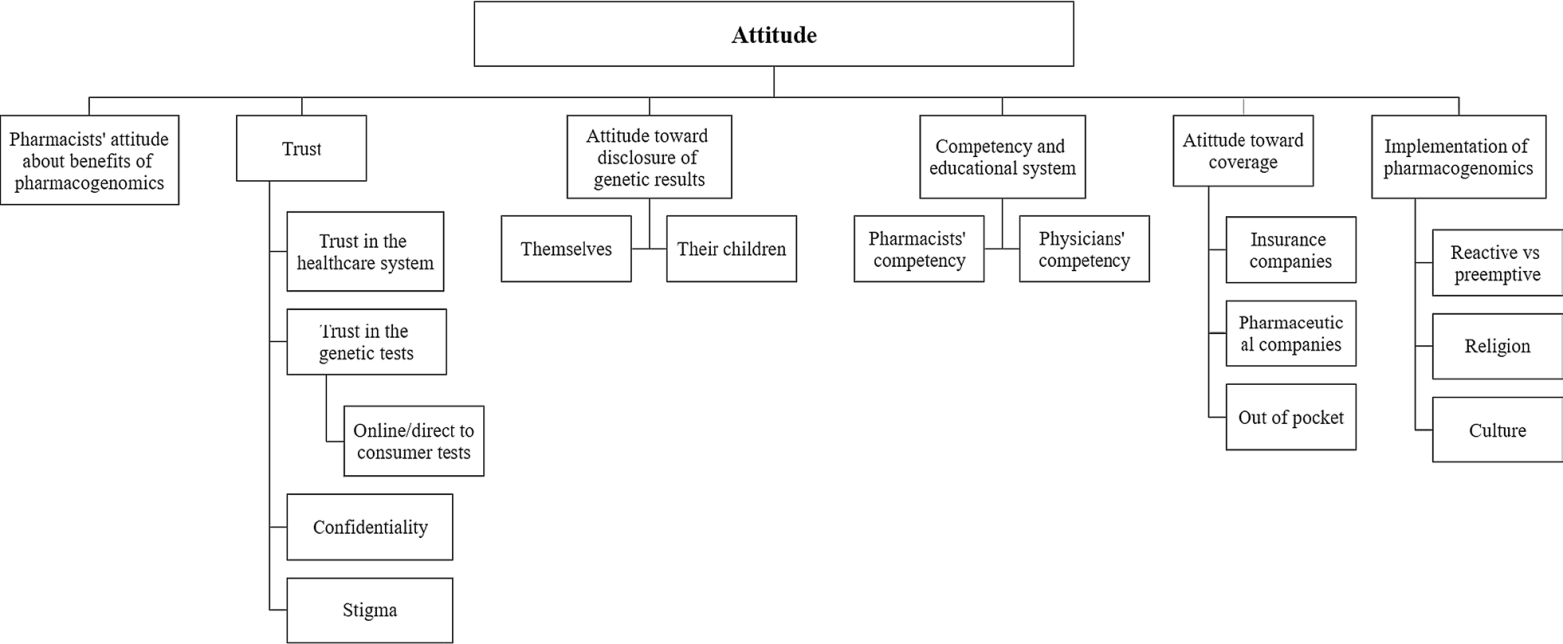
Themes and subthemes on the attitudes of pharmacists in the FGD

The main themes and subthemes of pharmacists’ PGx knowledge and attitudes extracted from the focus group discussions are shown in Figs. 1 and 2 respectively.

### Knowledge

Over a third of the participants perceived their current knowledge and understanding of PGx and genomics to be poor. They also reported having limited knowledge on where genetic testing is conducted in the UAE. When questioned on their higher education, both senior- and entry-level pharmacists stated they did not receive any formal education on PGx or genomics during their undergraduate pharmacy program. All clinical pharmacists noted a gap in the availability of resources by the UAE health authorities regarding information on genomics. Outpatient pharmacists were more familiar with the cost and accessibility of genetic testing.

### Attitudes

The majority of the pharmacists exhibited a positive attitude toward PGx despite their perceived lack of knowledge. However, there were some mixed responses on genetic testing. Some pharmacists were reluctant due to the possible discrimination and stigmatization associated with certain genetic diseases and the impact it could have on their social and family lives. Others viewed it as a protective measure allowing them to seek medical interventions and modify health behaviors in the case of a predisposition to a disease. Religious and cultural beliefs were noted as a potential barrier to the implementation of PGx in UAE.

When inquired on their skill and ability in interpreting genetic test reports, all pharmacists agreed they were not completely inept.

**Fig. 3:**
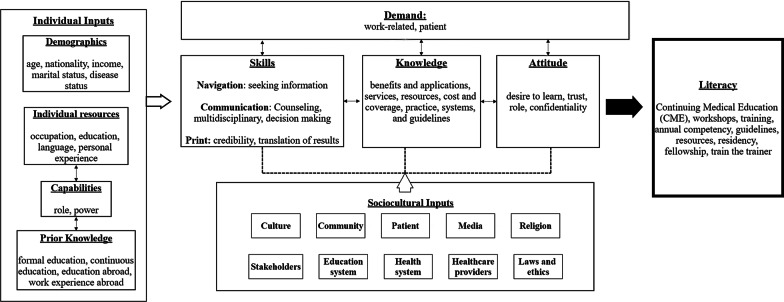
Pharmacogenomics/genomics literacy framework for pharmacists (PGLP)

The main themes of knowledge and attitudes constitute the main pillars of the PGLP framework which are consistent with the components of the HLS framework including: (1) the individual input of pharmacists, (2) demand for genomics’ literacy, (3) skills needed to improve illiteracy, (4) knowledge of the pharmacists about genomics/PGx and its applications, (5) attitudes of pharmacists and (6) sociocultural influences (Fig. 3).

## Discussion

The framework was centered on pharmacists since they are the core of PGx as articulated in the statement of The ASHP [12]. In this proposed PGLP framework, building PGx/genomics literacy for pharmacists depends on the following pillars:

### Individual inputs to literacy

Both the HLS and PGLP framework embrace the individual traits as input into literacy. An individual’s inputs like age, education, power, roles and capabilities are traits that need to be acknowledged and factored in while designing any training in any field and PGx is not an exception. One uniform approach has been abandoned and replaced by more tailored and personalized approaches that put the learner as the center and consider an individual’s inherited factors and capabilities to empower them [25, 32–35]. Many medical and health sciences colleges are embracing this evidence-based shift in paradigm by tailoring the pedagogy according to the learner’s individual traits [36–40].

In the PGLP framework, the diversity of pharmacists’ roles, power and capabilities is explored. For example, clinical pharmacists were more familiar with PGx than pharmacists in community settings. Also, pharmacists with children were keener to learn about PGx as they appreciate and foresee its value. Therefore, stakeholders planning a PGx workshop for pharmacists need to know their audience in regard to demographic, role, occupation, prior knowledge and experiences. We hypothesize that this will be a cost-effective PGx literacy approach [41–43]. A study by Owusu‐Obeng et al. explored the role of pharmacists in the PGx era and the findings aligned with ours. In their model, some of the individual inputs required from pharmacists were education, skills in informatics, background in medication safety, insight in medication‐use policies and procedures and conquest of literature assessment [13].

In the published PGx implementation models, in accordance with our findings, clinical pharmacists were appropriately situated to implement and lead clinical PGx programs, as they possess credible individual inputs such as prior knowledge and expertise in pharmacodynamics, kinetics, genomics, informatics and patient care [13, 44–48].

### Demand or stimuli

Our PGLP framework adopted the “demand” element from the HLS framework as it is the switch on button toward increasing genomic literacy and PGx implementation. The demand can originate from the patient and/or the clinical setting in a micro-, meso- and macro-level [21]. The ASHP statements highlighted the pharmacist’s patient-care loop. In their statement, patients were at the center stage for the demand for PGx implementation [12]. The demand is demonstrated by the interviewed pharmacists’ positive outlooks on genomic medicine and PGx despite their knowledge gap. Moreover, previous research shows that stakeholders emphasize clinical demand for genomic medicine in the UAE considering the high prevalence of consanguinity and high burden of genetic diseases [49–51].

### Skills

Critical skills of accessing, understanding, appraising and applying knowledge and information are an essential dimension of health literacy [30, 31, 52]. A study by Peterson‐Clark et al. [53] pointed out that pharmacists scored a wide-ranging but shallow general skills in surfing online information and e-health. A randomized clinical trial by Basheti et al. [54] reported that the retention of the skills of the pharmacists was significantly improved after training them on the proper technique of using inhalers and providing them with printed materials and tools. These findings are in congruence with our findings [27]. It is pivotal to include the “skills” element to the PGLP framework as electronic resources and databases, like Clinical Pharmacogenetics Implementation Consortium (CPIC) and PharmGKB, are the mainstay of PGx [55, 56].

### Knowledge

Knowledge of pharmacists is a profound repertoire of literacy. It was eluded in the HLS framework; however, we advocate and anchor its impact on health literacy in general and PGx/genomics in particular. Breadth of studies highlighted the PGx/genomics knowledge gap among pharmacists and other healthcare providers and the impact of this gap on implementation [22, 27, 57, 58].

In our PGLP framework, knowledge beyond an individual’s prior knowledge of PGx/genomics is emphasized. This encompasses knowledge of benefits and applications of PGx, available resources, services and practices, cost and insurance coverage and knowledge of the local, national and international guidelines. We foresee it as a dynamic pillar that needs to be addressed regularly by stakeholders planning literacy in PGx/genomics. Knowledge will speed the implementation and adoption of PGx/genomics in the practice setting of pharmacists. Pharmacists’ literacy and competency in PGx/genomics ought to be assessed and updated regularly [12, 22, 58–60]. Therefore, this component of the PGLP framework is vital. Credibility of the healthcare provider, in our case the pharmacists, has been quoted as being essential to the patient and community’s trust of any health information [31, 61, 62]. Thus, pharmacists’ knowledge of PGx will assert such trust from patients and community [27].

### Attitude

The HLS framework posed attitudes, feelings, incentive and self-worth as mediators between health literacy and outcome [31]. In our PGLP framework, attitude is stressed as an imperative cornerstone toward PGx literacy. Studies have shown that attitude of students, pharmacists or other healthcare providers has leverage on implementing PGx and genetic testing [41, 63–66].

Attitude and knowledge are additional pillars supplemented to improve literacy in PGx/genomics. We imputed a dynamic nature to skills as well as knowledge and attitude. Hence, these elements are interconnected and influence each other and influenced by the sociocultural inputs as well.

### Sociocultural influencers of literacy

The sociocultural determinants of our PGLP framework are more ample than the HLS framework as it incorporates ten inputs: culture, community, patient, media, religion, stakeholders, educational system, laws and ethics, health systems and healthcare providers. We conceptualize that these elements are cross-roads for PGx literacy. The World Health Organization (WHO) apprehended the same sociocultural factors that we appointed in our PGLP framework. Pang [67] named the fragile health care delivery systems as an obstacle to be addressed. Furthermore, WHO advises stakeholders to implement the following strategies to pursue PGx: effective communications, community trust, adopting a multidisciplinary approach to research, mounting ethical and regulatory frameworks, and involving all relevant stakeholders in decision- and policy-making.

Pharmacists are not isolated from the community, health system or other healthcare providers. Consequently, pharmacists in our cohort advocate a multidisciplinary approach to implement PGx. Studies favored this methodology. Caraballo et al. [68] employed a multidisciplinary task force of professionals to strike a balance in the implementation of PGx at the point of care. Another study by Dunnenberger et al. [69] concluded that a multidisciplinary PGx clinic can expedite the incorporation of PGx into clinical care.

In the literature, there is an aggregation of evidence of the PGx’s knowledge gap among healthcare providers in general [70–74] and pharmacists [27, 75, 76] in specific. Preponderance of literacy frameworks is dedicated to patients [30]; however, having a literacy framework for PGx dedicated for healthcare providers will systematically bridge the knowledge gap. The complexity and the multifactorial challenges of the health system coupled with the multidimensional aspects of health literacy necessitate a comprehensive framework to address literacy in PGxs [30, 77].

Assuring a competent healthcare workforce is one of the 10 Essential Public Health Services [78]. It encourages empowering all healthcare providers from all levels with ongoing knowledge. Literacy in PGx is challenged by the unprecedented advances in technology and research in the field coupled by the need of lifelong learning [26, 30, 47].

### How to use the PGLP framework?

This framework will guide stakeholders in their mission of equipping pharmacists, genetic counselors, doctors and nurses with skills required for the adoption and implementation of PGx. PGLP directs the attempts of stakeholders to educate pharmacists on PGx by taking into account their individual factors and tailoring modules to meet their roles, occupation and capabilities whether they are clinical, inpatient, outpatient or community pharmacists or pharmacists in a Pharmacy Therapeutic Committee (PTC) or Institutional Review Board (IRB).

Stakeholders occupied by implementing PGx in their countries should not isolate their approach from the sociocultural factors incubating and nourishing their infrastructure and resources. They have to tailor their map to their current educational system, health system and culture. They have to utilize media and call for laws and policy. Moreover, they need to factor in religion and literacy of the community. The demand for PGx/genomics literacy will kick-start PGx implementation resulting in educational efforts and utilization of the PGLP framework.

Catalyzing the three dynamic pillars of skills, knowledge and attitude of pharmacists or other healthcare providers will be a compelling formula for developing cost-effective personalized and profound modules and approaches. Knowing the pharmacists’ skills will guide the stakeholders in purchasing platforms, databases and other resources and will tailor orientation. Mapping the knowledge and attitude of the pharmacists will help shape the resources, workshops, seminars and competencies.

This PGLP framework is comprehensive and we theorize that it will tailor the implementation strategies in a standardized and systematic manner.

## Conclusion

In conclusion, we believe that our framework can be a guide for the implementation of genomic medicine and PGx among pharmacists as it addresses a plethora of individual and sociocultural factors in addition to the skills, demands, knowledge and attitudes of pharmacists. This framework guarantees a holistic bottom-up approach toward PGx implementation. Personalized medicine entails personalized efforts and frameworks. Similar frameworks can be created for other healthcare providers, patients and stakeholders.

The strength of our work is conceptualizing a novel, comprehensive and personalized PGx literacy theoretical framework tailored to pharmacists. Moreover, the PGLP framework is based on the validated theoretical HLS framework that was synthesized upon a number of literacy frameworks thus increasing the PGLP framework’s credibility [31]. Furthermore, knowledge and attitude were added as new pillars inherited with PGx literacy. Another strength is building the PGLP framework with mixed methods which added thoroughness and depth.

Moreover, this framework can be used to guide stakeholders in any country that are planning a strategy to implement PGx, as it is comprehensive and systematic. Additionally, this framework can be a platform to PGx literacy to other healthcare providers or even other health-related literacy. It is highly recommended to introduce new courses and training workshops for pharmacists to improve the chances of PGx application as knowledge is an essential and dynamic pillar for the implementation of genomic medicine and PGx. The glitch in navigations skills can be amended by the governing bodies by providing official clinical practice pathways and references for healthcare providers. The PGLP framework is a theoretical framework that needs to be validated. Future research on its implementation can validate this framework. We call researchers to test and validate this PGLP framework to pharmacists and extrapolate it to other healthcare providers.

## Data Availability

All data generated or analyzed during this study are included in this published article.
